# A Massive Renal Infarction Due to Atheroemboli: A Case Report

**DOI:** 10.7759/cureus.41842

**Published:** 2023-07-13

**Authors:** Long Nguyen Tuan, Yen Nguyen Thi Bach, Hung Nguyen Duc, Vu Nguyen Hoai

**Affiliations:** 1 Cardiology, Tam Anh Hospital, Ha Noi, VNM

**Keywords:** non valvular atrial fibrillation, embolic events, renal intervention, atheroemboli, renal artery infarction

## Abstract

The symptoms of acute renal infarction (ARI) caused by atheroemboli are vague, making it rare. Early diagnosis of renal infarction can be made through contrast-enhanced CT of the abdomen. However, diagnosing atheroemboli is more challenging. Kidney biopsy is the most accurate method to determine the cause, but it may not always be available in clinical settings. In cases where a thrombectomy is performed, white substances in thrombus aspiration or the patient's blood can suggest a diagnosis. Intervention is an effective technique, but there is controversy due to a lack of data, particularly in lobular artery infarction. We successfully treated one case using thrombus aspiration, and the specimens suggested atherosclerosis as the cause.

## Introduction

Renal infarction is a rare disease that often goes undiagnosed. According to Domanovits et al., only 0.007% (17/248,842) of people experience acute renal infarction (ARI) [1]. Failure to address this condition adequately can lead to severe health problems, including renovascular hypertension, chronic kidney disease, and end-stage renal disease. It is crucial to diagnose and treat ARI quickly to preserve renal function. Renal infarction symptoms are often ambiguous and can be mistaken for other health problems, potentially causing permanent damage to the renal parenchyma [2]. Contrast-enhanced CT scans and other imaging methods have led to more cases of renal infarction being identified in patients with non-specific symptoms.

Coagulation disorders, vasculitis, connective tissue diseases, valvular endocarditis, atherosclerosis, aortic aneurysms, smoking, and trauma can cause renal infarction [3]. It is uncommon for atherosclerosis emboli to occur, but it can lead to renal impairment by blocking small renal vessels and causing ischemia. Anticoagulants, vascular manipulation, or thrombolytic drugs may be linked to developing these emboli, but spontaneous cases also occur. The severity of atheroembolic renal disease can vary from mild or asymptomatic to life-threatening. The diagnosis process for this particular condition involves assessing clinical signs, risks, and contrasting scans, much like other diagnoses. However, a renal biopsy is necessary for an accurate diagnosis.

Additionally, there is currently no consensus on the optimal treatment for ARI due to a lack of literature on the subject. In this report, we share a case of spontaneous renal infarction caused by atherothrombosis. The diagnosis was made through specimens collected during thrombectomy and blood tests. Additionally, the treatment outcomes highlight the efficacy of thrombus suction in treating infarction in the lobular artery.

## Case presentation

A 52-year-old male arrived at our hospital with acute left-sided flank pain extending toward the hypochondrium. The symptom began 21 hours before his admission, and there is no history of fever, jaundice, constipation, diarrhea, burning micturition, hematuria, trauma, drug intake, alcohol intake, or weight loss. The patient has a medical history of smoking for 30 pack years and chronic gout, but there is no record of atrial fibrillation, diabetes mellitus, hypertension, or previous renal failure. Furthermore, there are no reported instances of these conditions in the patient's family history.

The patient's vital signs include a body temperature of 36.2°C, blood pressure of 189/100 mmHg, pulse rate of 88 bpm, respiratory rate of 18 breaths/min, and oxygen saturation of 97%. The cardiac and respiratory examinations showed no abnormalities. The abdomen was soft and not swollen, moved with respiration, and had no tenderness, guarding, or rigidity. Moreover, there was no tenderness or discomfort upon palpation of the kidney and signs of organ enlargement or free fluids in the abdomen. Bowel sounds were normal, and the urine output was sufficient.

Table 1 displays the metabolic evaluation results of the patient.

**Table 1:** Laboratory parameters of the patient PCI: percutaneous coronary intervention, RBC: red blood cell, HPF: high-power field, CRP: C-reactive protein

Laboratory parameters	Pre PCI	Post PCI	One-month follow-up
Hemoglobin (g/dl)	14	13.5	12.7
White blood cells (G/L)	13500	25000	4600
Serum urea (mmol/L)	4.4	4.9	5.3
Serum creatinine (µmol/L)	135	96	98
Creatinine clearance (ml/ph)	48	68	66
Urine microscopy (RBC/HPF)	10	7	negative
Uric acid (µmol/L)		614	232
Glucose (mmol/l)	6.4	6.2	8.2
CRP (mg/L)	16.1	25.1	0.28
Cholesterol (mmol/l)		3.8	2.6
Triglyceride (mmol/l)		2.41	1.95
Blood pressure (mmHg)	189/100	130/80	130/70

The examination found the absence of left anterior segmental arterial flow during an abdominal ultrasound with renal Doppler. The ECG showed a normal sinus rhythm without any evidence of arrhythmias, and the echocardiography did not reveal any abnormal wall motion or vegetation within the cardiac chambers.

**Figure 1:**
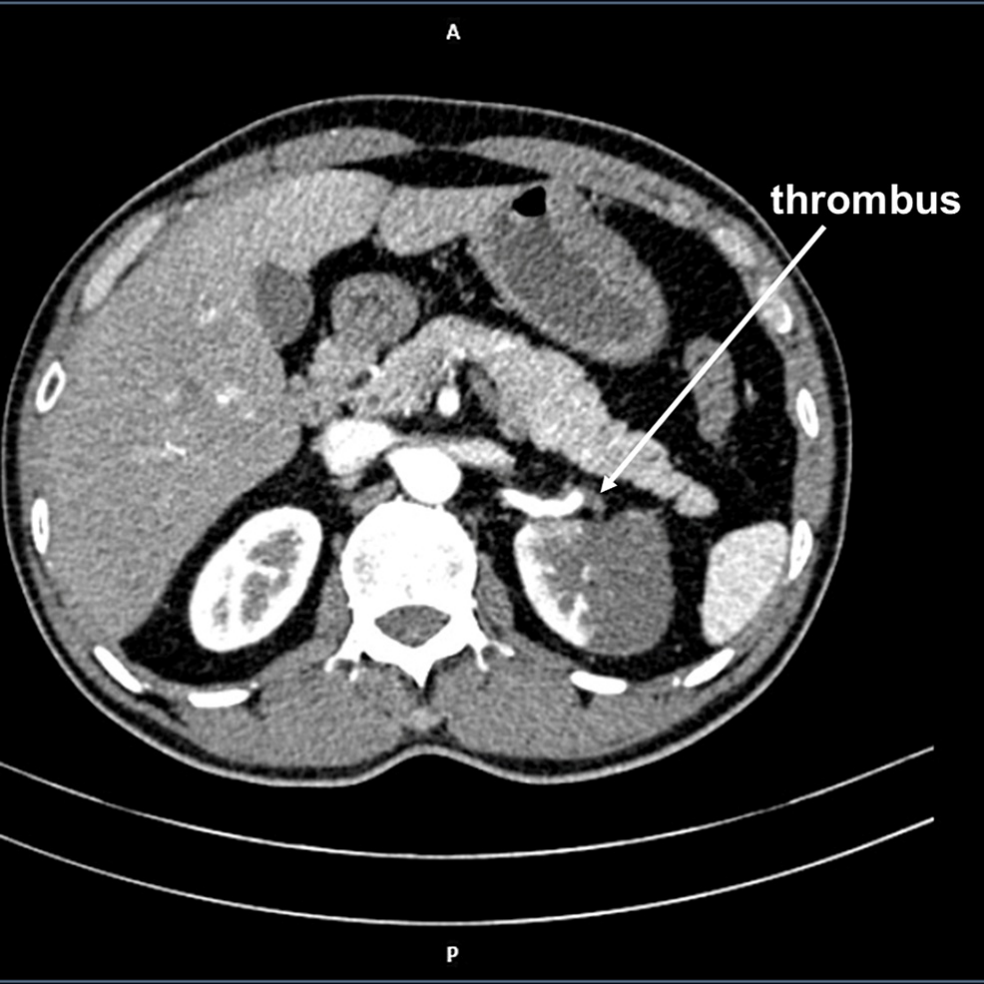
Contrast-enhanced CT scan of the abdomen showed an area of non-enhancement in the left kidney and a thrombus in the anterior segment artery

A contrast-enhanced CT scan of the abdomen was performed, which showed an area of non-enhancement in the left kidney and a thrombus in the anterior segment artery (Figure 1).

Because of persistent symptoms of ischemia duration of the pain below 24 hours, we considered thrombectomy therapy. Therefore, the patient was transferred to the catheterization laboratory one hour after the diagnosis.

**Figure 2:**
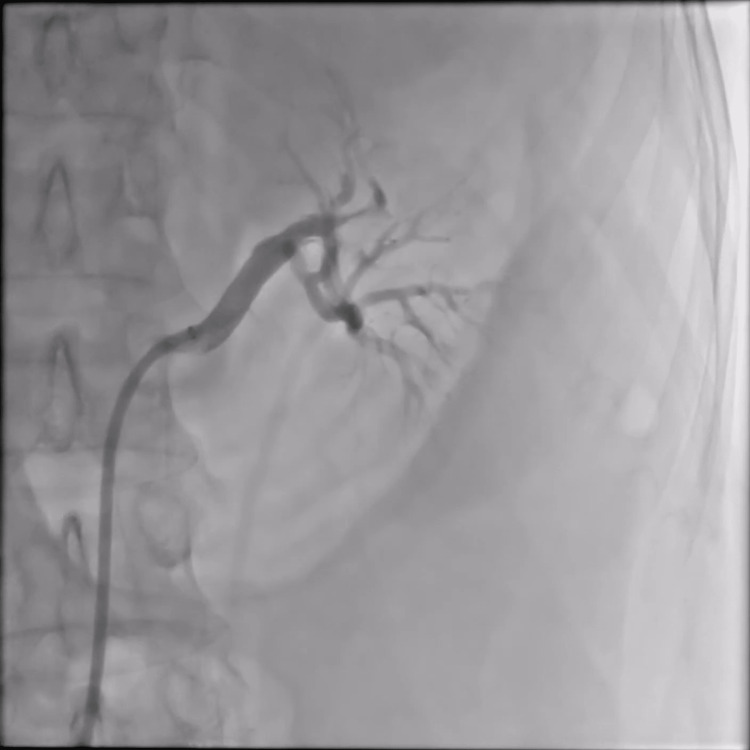
Angiography showing occlusion of two segment branches of the left renal artery

The result of the angiography showed no-flow perfusion in the left renal artery (Figure 2).

**Figure 3:**
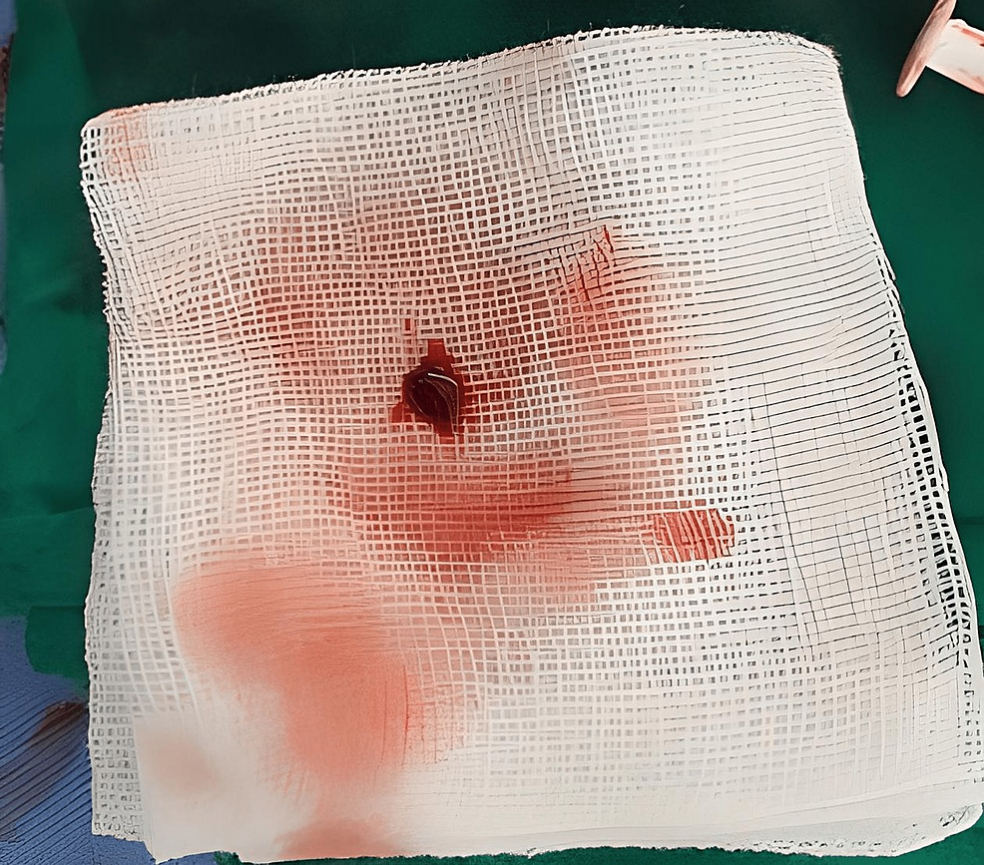
Red thrombus was aspirated from the left renal artery

**Figure 4:**
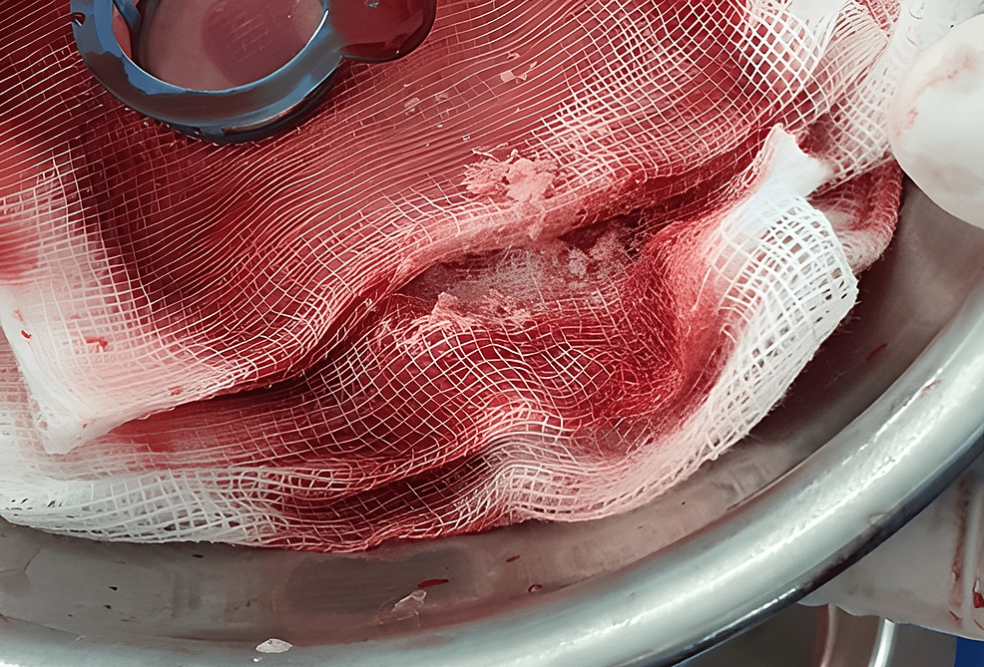
White tissue that aspirated from the left renal artery

**Figure 5:**
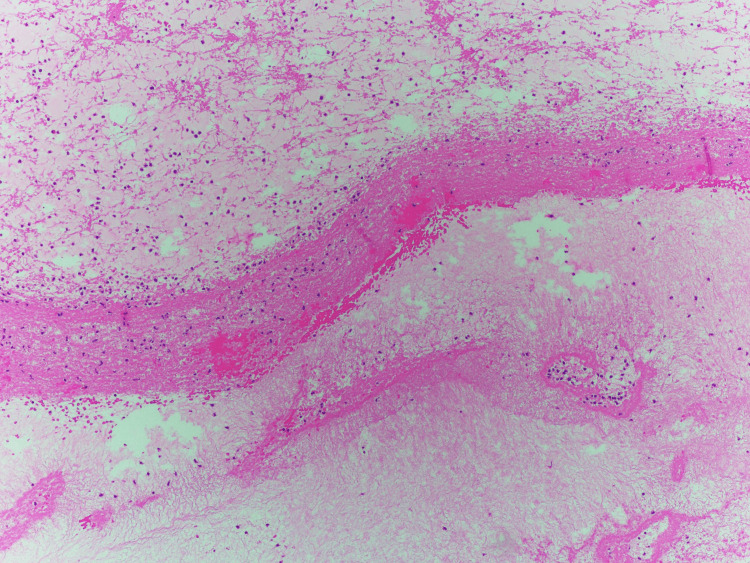
Pathophysiology showing the thrombosis (proteins and lipids and red and white blood cells) incompletely degraded (no tumor cells were seen)

Our first attempt at direct thrombectomy using the guiding JR proved unsuccessful due to a thrombus in the segmental arm. We had to alter our approach and use the Solumbra system (Straub Medical, Wangs, Switzerland) to suction out the thrombus instead. Eventually, we extracted some thrombus (Figure 3) and white matter of unknown origin (Figure 4). Then, the left artery was almost completely reperfused (Figure 5).

**Figure 6:**
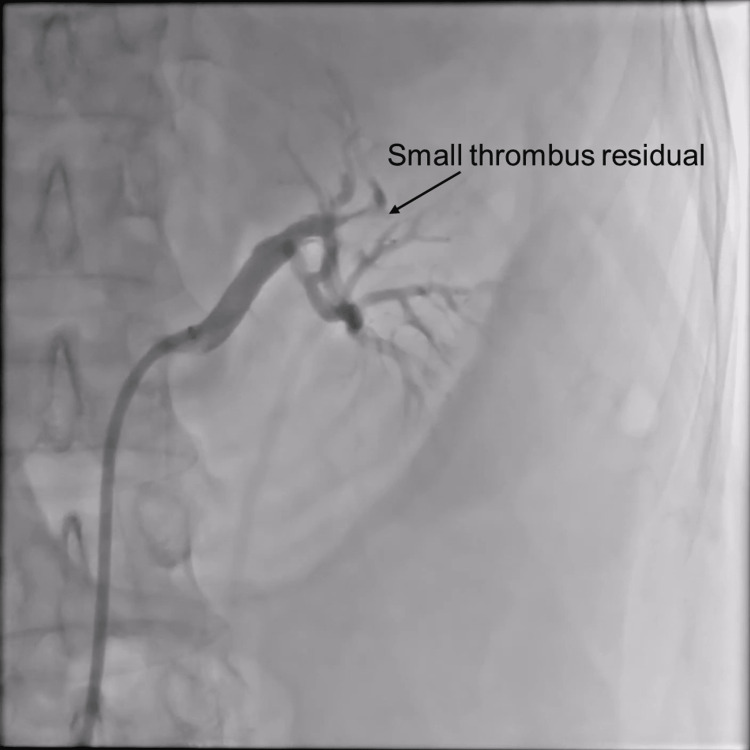
Partial recanalization of the renal artery has been observed after thrombus aspiration. Nonetheless, a small lobular artery still contains a small amount of thrombus

Figure 6 shows that the left artery appears to be nearly done with reperfusion. However, a small segment of the artery still has some thrombus. As a result, we decided to halt the procedure.

**Figure 7:**
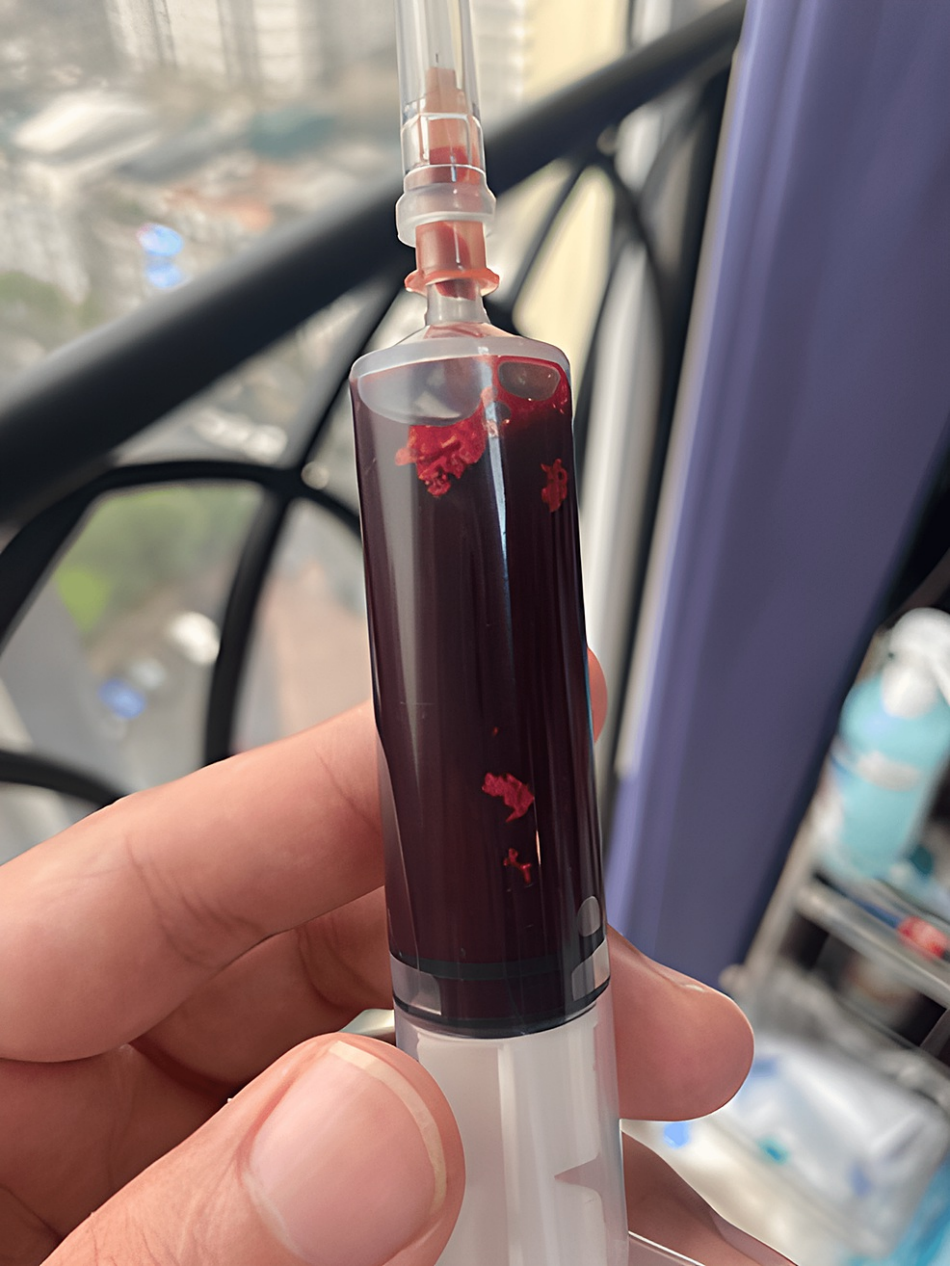
The blood sample showing many white substances

The patient's pain was alleviated with oral analgesics, which were stopped after one day. Then, we were given a full dose of low-molecular-weight heparin (enoxaparin 1 mg/kg 12 hourly) to start anticoagulation, which was continued for five days. Warfarin was initiated on day one and overlapped with enoxaparin for five days to achieve a target international normalized ratio of 2-3. However, after five days, the patient's renal function returned to normal (creatinin 85 µmol/L). Therefore, warfarin was switched to apixaban 2.5 mg BID. Three days after, we increased apixaban to 5 mg BID. Extensive metabolic testing was performed to identify any risk factors or hypercoagulable states. Still, no abnormalities were detected in ANA, protein S, protein C, ATIII, factor V Leiden, prothrombin 20210A mutation, antiphospholipid antibodies, or homocysteine levels. The blood culture was negative, and the white tissue sample collected from the patient's blood (figure 7) showed a badly degenerated thrombus. Transesophageal echocardiography revealed no tumors or thrombi in the patient's heart. The patient was discharged five days later on apixaban.

## Discussion

ARI is a rare disease caused by a blockage in the renal arteries. Individuals with ARI may experience sudden abdominal or flank pain and symptoms such as nausea, vomiting, hematuria, and sometimes fever. Abdominal or loin tenderness may also occur during examination. A retrospective study of 94 patients showed that 96.8% had abdominal pain, 27.6% experienced nausea, and 20.2% presented with vomiting and fever. The study also found that 48% of patients had hypertension [4]. However, the clinical symptoms are not specific, which could lead to a delayed diagnosis if overlooked by the doctor. The mechanism of hypertension is the augmentation of the renin reaction after the loss of the renal artery, which can be mistaken for a pain response. Mesiano et al. found that patients with renal infarction had an average systolic arterial pressure of 147.7 +/- 18.2 mmHg and an average diastolic arterial pressure of 83.2 +/- 9.5 mmHg [5]. Upon admission to the hospital, our patient was diagnosed with hypertension, but their blood pressure decreased within a day.

Renal infarction can result from various factors such as coagulation disorders, vasculitis, connective tissue diseases, valvular endocarditis, atherosclerosis of the renal artery or aorta, smoking, and trauma. It can also be caused by aeroembolism [6]. We are testing to determine the event's cause, but the results thus far have not provided any clear answers. However, upon examining the aspirated thrombus, we observed numerous white tissues that indicate the possibility of atheroembolic reason (Figure 4). One day after, when we took blood for laboratory tests, we saw many white tissues (Figure 7). With that observation, we have proof to think about atherosclerosis.

Atheroembolic renal disease is a clinical condition that is often underdiagnosed. It may also be called cholesterol atheroembolic renal disease, atheroembolism, cholesterol embolism, or cholesterol crystal embolization [3]. Cholesterol emboli can cause kidney damage by blocking small blood vessels due to atherosclerosis, leading to inflammation and giant cell formation. Anticoagulants, vascular manipulation, or thrombolytic drugs can sometimes be linked to these emboli, but spontaneous cases have also been documented [7]. Symptoms can range from mild to life-threatening, including acute renal failure. Symptoms of atherosclerotic emboli causing renal infarction may include eosinophilia, eosinophiluria, and low serum complement, but these usually disappear within a week [8]. Atheroembolic renal emboli can also slowly reduce renal function over three to eight weeks and cause neurological defects, mental confusion, localized cyanosis, and retinal Hollenhorst plaques [9,10].

To diagnose embolic renal infarctions, a renal biopsy may be needed. This procedure has a success rate of over 75% in detecting other emboli, but only 12% of cases are currently diagnosed correctly and promptly [6]. The biopsy may show bi-convex or needle-shaped defects, or "ghosts," and reveal significant vascular intimal inflammation, eosinophilic infiltration, and focal glomerulosclerosis. Intraluminal cholesterol crystals may dissolve during the fixation process [3,11]. However, a kidney biopsy may not always be possible. Upon pathological examination, the presence of white substances and inadequate degradation of the thrombus may indicate that the renal infarction was caused by atherosclerotic plaque. This is unlikely a white thrombus as they typically have a high concentration of platelets [12].

When dealing with patients with renal infarction, it is crucial to assess their risk factors thoroughly. Risk factors are smoking history, hypercholesterolemia, hypertension, and male gender; for older males with a history of cardiac issues and hypertension, as well as renal complications but no signs of infection or stones, ARI may be possible [12]. An abdominal contrast CT is recommended to confirm the diagnosis, which may reveal a hypodensity or wedge-shaped perfusion defect. During an intravenous contrast-enhanced CT, the cortical rim sign may be observed in about half of the renal infarction cases [13].

If someone suspects renal infarction, renal CT arteriography is the most reliable way to diagnose it and make informed treatment decisions. Although renal MRI arteriography can be helpful, it takes much longer to perform; therefore, CT imaging is typically preferred [14].

Treatment options for RAT include anticoagulation, percutaneous interventional therapy, and surgery [6]. Anticoagulants alone may not be enough to relieve symptoms and renal dysfunction resulting from renal infarcts; therefore, endovascular treatments like local intra-arterial thrombolysis, catheter aspiration, balloon dilatation, and stent placement are effective in restoring blood supply and preventing loss of renal function and hypertension. Quick diagnosis and treatment are crucial, as irreversible renal injury can occur within three hours of symptom onset [15]. Revascularization procedures are assessed based on factors such as time since onset of ischemia, current kidney function, size and extent of the renal infarct, presence of arterial dissection, the function of the contralateral kidney, precise vessels involved, and whether the occlusion of the renal artery is partial or complete.

Certain conditions increase the likelihood of effective revascularization, including complete arterial blockage lasting less than six hours, having only one kidney, or a significant decrease in renal function with a glomerular filtration rate of less than 50 mL/min from baseline [6]. It is crucial to consider an artery, even if it has been partially obstructed for fewer than 24 hours. Still, if the blockage has lasted longer than 24 hours, it should only be considered if the patient experiences renal failure, persistent flank pain, or new/worsening hypertension [16]. Revascularization may also be necessary in cases of renal infarction associated with arterial dissection [17].

We performed a thrombectomy intervention due to a complete blockage of the vital renal artery causing persistent left flank pain. We used the Solumbra technique to reopen the blocked artery, and the pain subsided completely. After the procedure, there was a transient increase in the creatinine level, but the affected side's renal function improved somewhat after two months.

## Conclusions

Atherothrombotic renal infarction is a condition that can have various causes, including unknown ones, and is considered rare. Diagnosis is based on clinical factors and suggestive signs, with abdominal CT used to confirm it. The presence of atherosclerotic plaques circulating in the blood or aspiration thrombus at the occlusion site is highly suggestive of the disease. Thrombosis intervention can help improve clinical symptoms if the patient remains symptomatic, even if it is only an infarction in the lobular arteries. To assess the effectiveness of long-term treatment results, more research studies are necessary.
